# Caffeine Modulates Cadmium-Induced Oxidative Stress, Neuroinflammation, and Cognitive Impairments by Regulating Nrf-2/HO-1 In Vivo and In Vitro

**DOI:** 10.3390/jcm8050680

**Published:** 2019-05-14

**Authors:** Amjad Khan, Muhammad Ikram, Tahir Muhammad, Junsung Park, Myeong Ok Kim

**Affiliations:** Division of Applied Life Science (BK 21), College of Natural Sciences, Gyeongsang National University, Jinju 52828, Republic of Korea; amjadkhan@gnu.ac.kr (A.K.); qazafi417@gnu.ac.kr (M.I.); mtahir.khan@gnu.ac.kr (T.M.); jsp@gnu.ac.kr (J.P.)

**Keywords:** cadmium, caffeine, reactive oxygen species (ROS), Nrf-2/HO-1, memory impairments, p-NF-κB, neurodegeneration

## Abstract

Cadmium (Cd), a nonbiodegradable heavy metal and one of the most neurotoxic environmental and industrial pollutants, promotes disturbances in major organs and tissues following both acute and chronic exposure. In this study, we assessed the neuroprotective potential of caffeine (30 mg/kg) against Cd (5 mg/kg)-induced oxidative stress-mediated neuroinflammation, neuronal apoptosis, and cognitive deficits in male C57BL/6N mice in vivo and in HT-22 and BV-2 cell lines in vitro. Interestingly, our findings indicate that caffeine markedly reduced reactive oxygen species (ROS) and lipid peroxidation (LPO) levels and enhanced the expression of nuclear factor-2 erythroid-2 (Nrf-2) and hemeoxygenase-1 (HO-1), which act as endogenous antioxidant regulators. Also, 8-dihydro-8-oxoguanine (8-OXO-G) expression was considerably reduced in the caffeine-treated group as compared to the Cd-treated group. Similarly, caffeine ameliorated Cd-mediated glial activation by reducing the expression of glial fibrillary acidic protein (GFAP), ionized calcium-binding adapter molecule 1 (Iba-1), and other inflammatory mediators in the cortical and hippocampal regions of the mouse brain. Moreover, caffeine markedly attenuated Cd-induced neuronal loss, synaptic dysfunction, and learning and cognitive deficits. Of note, nuclear factor-2 erythroid-2 (Nrf-2) gene silencing and nuclear factor-κB (NF-κB) inhibition studies revealed that caffeine exerted neuroprotection via regulation of Nrf-2- and NF-κB-dependent mechanisms in the HT-22 and BV-2 cell lines, respectively. On the whole, these findings reveal that caffeine rescues Cd-induced oxidative stress-mediated neuroinflammation, neurodegeneration, and memory impairment. The present study suggests that caffeine might be a potential antioxidant and neuroprotective agent against Cd-induced neurodegeneration.

## 1. Introduction

Heavy metals, which have been used by humans for decades, present toxic health hazards to the human population and the environment. They affect human health and the surroundings in several ways, by polluting the air, water, and soil. The seven most common heavy metals are cadmium (Cd), zinc (Zn), mercury (Hg), nickel (Ni), lead (Pb), chromium (Cr), and copper (Cu), all of which affect humans and the environment [1,2,3]. Among them, Cd is a widespread and nonbiodegradable environmental pollutant that has a broad range of adverse effects on humans. Cd has a half-life of about 7–30 years and cannot be degraded by microorganisms, so it easily enters the food chain through cosmetics, fertilizers, paints, automobiles, industrial workplaces, batteries, and cigarette smoke, leading to accumulation of Cd in the ecosystem. 

Cd is accumulated in vital human organs including the brain, heart, liver, kidneys, lungs, and bones and causes structural and functional destruction [4,5,6]. Among these organs, the brain is the main target for Cd-induced neurotoxicity as it increases the permeability of the blood–brain barrier (BBB) and induces oxidative stress, neuroinflammation, and behavioral deficits [7,8]. The mechanism of Cd-induced neurotoxicity is not well documented; however, several studies have shown that oxidative stress is the main contributor to the pathogenesis of Cd-induced neurodegeneration and memory impairment. Oxidative stress is responsible for mitochondrial membrane disruption and results in reduced synthesis and release of adenosine triphosphate (ATP). Cd also alters the activity of enzymes that neutralize oxidative stress [9,10,11]. Several studies have reported that Cd promotes reactive oxygen species (ROS) generation and lipid peroxidation (LPO) production in the brain. Due to high oxygen consumption and the low activity of antioxidant defenses, the central nervous system (CNS) is highly susceptible to oxidative stress. Nuclear factor-2 erythroid-2 (Nrf-2) is the main player in the endogenous antioxidant system, playing a key role against ROS and upregulating other endogenous genes such as hemeoxygenase-1 (HO-1), which neutralizes ROS and LPO and has a protective effect against oxidative stress-induced neurodegeneration [12,13]. It has also been reported that neuronal oxidative stress is known to activate the proinflammatory mediators and transcription factors, causing neuroinflammation and neurodegeneration [14,15]. Cd is responsible for impairment of the neuronal antioxidant defense system, causing neuronal death and apoptosis, leading to neurodegeneration and memory impairment [16].

Caffeine, widely found in coffee, black and green tea, soft drinks, and energy drinks, is the most consumed psychostimulant. Generally, coffee beans contain 10–12 mg/gm of caffeine. After oral ingestion, it is absorbed quickly from the gastrointestinal tract, and the highest concentration of caffeine is reached after 30–60 min. It is also reported that caffeine easily crosses the BBB due to its hydrophobic nature, and similar concentrations are found in the brain as in the blood [17,18,19]. Caffeine increases alertness and reduces fatigue, which leads to better performance, reduces the number of driving incidents, and improves memory and learning [20,21]. Moreover, studies have also reported that caffeine exerts neuroprotective effects against various neurodegenerative disorders [22,23], is a potent reactive oxygen species (ROS) scavenger, and improves cognition in the rat brain by altering the endogenous antioxidant system [23,24].

In this study, we hypothesized that Cd induces oxidative stress, neuroinflammation, synaptic dysfunction, and memory impairment. Based on its antioxidant effects, we used caffeine as a potential neuroprotective agent. We confirmed the anti-inflammatory, cytoprotective, and nootropic effects of caffeine in an adult mouse model with HT-22 and BV-2 cell lines.

## 2. Material and Methods

### 2.1. Chemicals

Cadmium chloride and caffeine were purchased from Sigma-Aldrich Chemical Co. (St. Louis, MO, USA).

### 2.2. Animals

Wild-type C57BL/6N male mice (*n* = 45, 8 weeks old, 25–30 g weight) were purchased from Samtako Bio (Osan, South Korea). All mice were adapted for 1 week under 12-hour light/dark conditions at 23–25 °C with 60 ± 10% humidity and were provided with free access to water and food in the university animal house. All procedures were approved and conducted in accordance with the guidelines of the animal ethics committee of the Division of Applied Life Sciences, Gyeongsang National University, South Korea (approval ID: 125).

### 2.3. Drug Treatment

**Figure 1:**
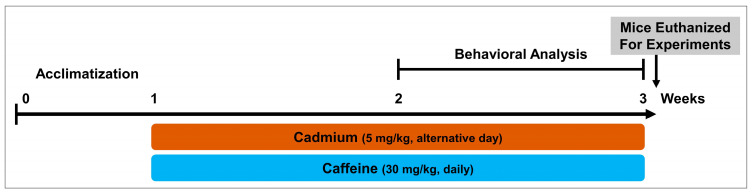
Schematic diagram of experimental design showing duration of cadmium (as cadmium chloride) and/or caffeine treatment in adult mice and their behavioral analysis.

The mice were randomly placed into three groups and treated as follows (Figure 1):

(1) Control mice treated with saline as a vehicle for 2 weeks (intraperitoneal, IP).
(2) Mice treated with Cd chloride 5 mg/kg, alternative day as a neurotoxic agent for 2 weeks (IP).
(3) Mice treated with Cd chloride 5 mg/kg/day and caffeine 30 mg/kg/day for 2 weeks (IP).

### 2.4. Behavior Study

The Morris water maze (MWM) test was used to evaluate spatial learning and memory functions as described previously with some modification [25,26]. The apparatus consisted of a circular water tank 40 cm in height, 100 cm in diameter, and 15.5 cm deep, containing water (temperature 23 ± 1 °C), that was rendered opaque by adding white ink. At the midpoint of one quadrant, a hidden platform 10 cm in diameter and 20 cm in height was placed 1 cm below the water surface. After the mice received 2 consecutive days of training, latency was calculated (in seconds) to assess the time taken to reach the hidden platform over 4 consecutive days. One day after the training session, a probe test was performed by removing the hidden platform and allowing each mouse to swim freely in the water tank for 60 s to evaluate their memory. The number of times crossing over the previously hidden platform and the time spent were measured for each mouse in the quadrant where the hidden platform had been present. All data were recorded using video-tracking software.

For Y-maze analysis, each mouse was allowed to move freely in the center of the apparatus for 8 min. The series of arm entries was recorded digitally. Spontaneous alteration was defined as the successive entry of a mouse into three arms in overlapping triplet sets. The altered behavior percentage (%) was calculated as (successive triplet sets (entry into three arms consecutively)/total number of arm entries − 2) × 100. Improved memory and cognitive function were reflected by a higher percentage of spontaneous altered behavior.

### 2.5. Protein Extraction

After behavior analysis, all animals were anesthetized with ketamine/xylazine and brains were removed, and the cortex and hippocampus were immediately separated and stored at −80 °C. Cortex and hippocampus were homogenized in PRO-PREP (a protein extraction solution). The samples were then centrifuged at 13,000 rpm at 4 °C for 30 min, and the supernatants were collected and stored at −80 °C.

### 2.6. ROS and LPO Assays

ROS and LPO assays were performed as described previously with some modifications [27]. The principle of ROS assay is the formation of 2′7′ dichlorofluorescein (DCF) from the oxidation of 2′7′-dichlorodihydrofluorescein. In ice-cold buffer, the cortex and hippocampus homogenates were diluted to yield 2.5 mg tissue/500 μL as a final concentration. Then, 1 mL of Locke’s buffer mixture (pH ± 7.4), 0.2 mL of homogenates, and 10 mL of 5 mM 7-dichlorodihydrofluorescein diacetate (DCFH-DA) were incubated for 15 min at room temperature to form fluorescent DCF from DCFH-DA. The formation of DCF from DCFH-DA was evaluated via a microplate reader at 484 nm (excitation) and 530 nm (emission). In the absence of homogenate, to enable calculation of DCF formation (background fluorescence), we measured parallel blanks. ROS levels are expressed as DCF formed (pmol)/amount of protein (mg). For further determination of oxidative stress, the level of LPO was quantified. Cortex and hippocampus of adult mice were homogenized and free malondialdehyde (MDA), a specific marker for LPO, was measured with a colorimetric/fluorometric assay kit according to the manufacturer’s instructions (BioVision, San Francesco, CA, USA, Cat#739-100).

### 2.7. In Vitro Cell Culture

**Figure 2:**
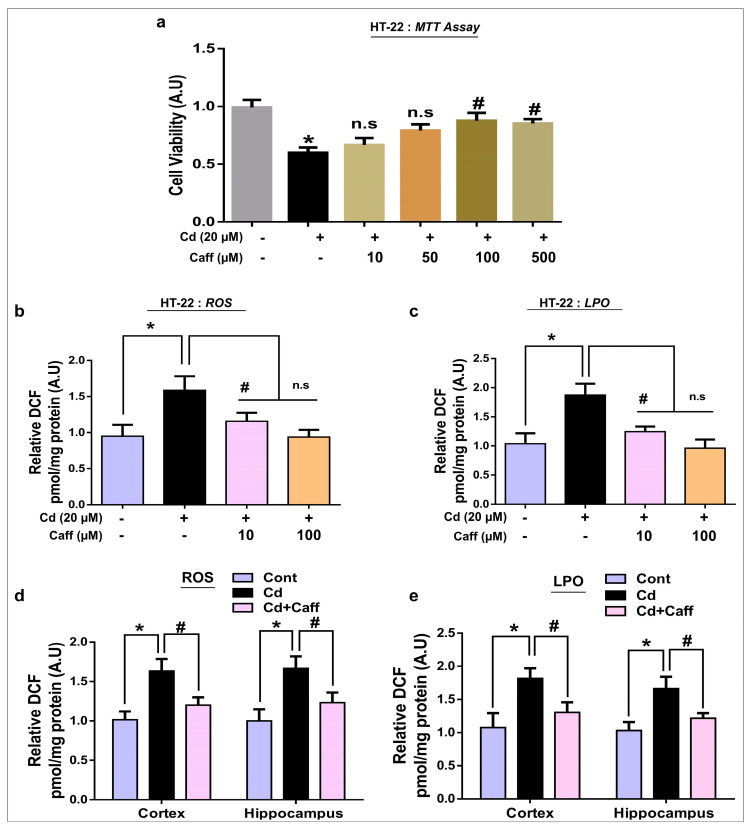
The protective effects of Caffeine on cell viability, reactive oxygen species (ROS) and lipid peroxidation (LPO) in Cd-treated HT-22 cells. (**a**) The MTT (a cell viability) assay in HT-22 cells showing the cell viability in various treated groups, (**b**,**c**) Analysis of the generation of ROS and production of LPO in HT-22 cells. (**d**,**e**) Analysis of the ROS generation and LPO production in the cortex and hippocampus regions of mice brain. The data are presented as the mean ± SEM of triplicate wells for in vitro and of 6–8 mice per group and are representative of three independent experiments, magnification 10×, scale bar = 50 µm and the expressed data are relative to the control (saline-treated). ⁎ significantly different from the vehicle-treated group; # significantly different from Cd-treated group. Significance = ⁎ *p* < 0.05, # *p* < 0.05.

Mouse hippocampal (HT-22) and murine microglia (BV-2) cell lines were cultured in Dulbecco’s modified Eagle’s medium (DMEM) supplemented with 10% fetal bovine serum (FBS) and 1% antibiotic/antimycotic in an incubator supplied with 5% CO_2_ at 37 °C. After gaining 70% confluency, cells were treated with Cd (1 μg/mL) and caffeine (100 µM), and the dose of caffeine that significantly attenuated Cd toxicity was selected (Figure 2a), as well as Nrf-2 small interfering RNA (siRNA) or BAY 11-7082 (15 μM) for specific inhibition of phosphorylated-nuclear factor-κB (NF-κB). All chemicals/drugs were treated and incubated for 24 h at 5% CO_2_ and 37 °C.

### 2.8. Cell Viability Assay

To assess the viability of cells, 3-(4,5-dimethylthiazol-2-yl)-2,5-diphenyltetrazolium bromide (MTT) assay was performed as previously described [28]. In 96-well plates, cells were cultured at a density of 1 × 104 cells/well containing 100 μL of DMEM. After the attachment of cells, the medium was replaced with fresh medium containing Cd (1 μg/mL) and/or 10, 50, 100, or 500 µM concentration of caffeine. After incubation of cells for 24 h, followed by incubation with MTT solution for 4 h, followed by addition of 100 μL of dimethylsulfoxide (DMSO) per well, the plate was shaken for 10–30 min. Absorbance was measured via microplate reader at 570 nm, repeated three times. 

### 2.9. In Vitro Nrf-2 Gene Silencing by siRNA

HT-22 neuronal cells were cultured in 75 cm^2^ flasks (Nunc™ EasYFlask™, Thermo Fisher Scientific, Nunc a/S, Rosklide, Denmark). Cells were counted by using a disposable hemocytometer followed by the addition of 10 μL of medium containing cells on both sides of the hemocytometer chamber. The middle central square and four 1/25 mm^2^ corners were counted by using an Olympus microscope under 10× magnification. The cells (2 × 10^4^/mL) were further subcultured in 35-mm Petri dishes (Thermo Fisher Scientific, Nunc a/S, Rosklide, Denmark) in DMEM supplemented with 10% FBS and 1% antibiotic at 37 °C in humidified air containing 5% CO_2_ according to the manufacturer’s protocol (SC: 37049, Santa Cruz Biotechnology, Inc. Dallas, TX, USA) for 36 h. The Nrf-2 was knocked down via Nrf-2 siRNA at a concentration of 10 μM/transfection. Negative siRNA (Ambion, Austin, TX, USA) was used as a control and lipofectamine™ 2000 reagent (Invitrogen) was used for transfection when the culture was 75–80% confluent. After 36 h, cells were exposed to Cd (1 μg/mL) and/or caffeine (100 μM), and in the control group to 0.01% DMSO for 24 h. After that, cells were collected in 1% PBS solution and centrifuged, followed by the removal of supernatant. Proteins were isolated by dissolving the pellets in protein extraction solution, then vertexed and ultrasonicated, and processed for Western blot analysis.

### 2.10. In Vitro LPO and ROS Assays

HT-22 cells were cultured in a 96-well plate containing 200 μL of DMEM medium per well at 37 °C for 24 h in a humidified incubator with 5% CO_2_. After 24 h, the medium was replaced with fresh medium containing Cd (1 μg/mL) and/or caffeine (100 μM). For ROS analysis, cells were exposed to DCFH-DA (50 μM) at 37 °C for 30 min. Absorbance for ROS-positive cells was measured at 484/530 nm. LPO level was assessed as described for the in vivo experiment. 

### 2.11. Antibodies

All primary antibodies used in this study are given in Table 1.

### 2.12. Western Blot Analysis

Western blot was performed as described previously with some modification [29,30,31]. Briefly, protein (15–30 μg) was run through 12–15% SDS-PAGE gel electrophoresis along with the marker (GangNam-STAIN, iNtRON Biotechnology, Burlington, NJ, USA) and then transferred to polyvinylidene-difluoride (PVDF) membranes. Membranes were blocked using 5% skim milk to reduce nonspecific binding and incubated with primary antibodies (1:1000 dilution) at 4 °C overnight. Immunoreaction was detected using EzWestLumi-One (Amersham ECL Advance Western Blotting Detection reagent) and luminescence was recorded via X-ray film. The X-ray films were scanned and measured by ImageJ software. Similarly, HT-22 and BV-2 cells were collected in 1% PBS solution, centrifuged, and the supernatant was removed. The remaining pellets were dissolved in PRO-PREP solution by vortexing and ultrasonication for Western blot analysis.

### 2.13. Tissue Sample Preparation for Morphological Analysis

After the completion of treatment, mice were transcardially perfused with 0.9% normal saline solution and 4% paraformaldehyde. Brains were removed and fixed with ice-cold paraformaldehyde at 4 °C for 72 h, and then sunk into 20% sucrose phosphate buffer for 72 h. All brains were frozen in O.C.T. Compound (Sakura Finetek USA, Inc., Torrance, CA, USA) and 14-μm brain sections were taken by using a CM 3050C cryostat (Leica, Germany). The sections were thaw-mounted on gelatin charged slides (Fisher, Rock-ford, IL, USA).

### 2.14. Immunofluorescence Staining

Immunofluorescence was performed as described previously with some modification [32,33]. Slides with brain sections were washed twice with 1% PBS for 10 min, then proteinase-k was added, followed by incubation for 5 min at room temperature. All slides containing brain sections were washed twice for 5 min and then incubated for 1 hour with a blocking solution containing 2% normal serum and 0.3% Triton X-100 in 1% PBS according to the antibody used. All slides were incubated with primary antibodies (1:100) at 4 °C overnight. After incubation with primary antibodies, the brain sections were incubated for 2 h with tetramethyl rhodamine isothiocyanate (TRITC)/fluorescein isothiocyanate (FITC)-labeled secondary antibodies (1:50) (Santa Cruz Biotechnology). Incubation with antibodies was followed by the addition of 4, 6-diamidino-2-phenylindole (DAPI) for 8 min, mounted with DPX (Distyrene Plasticizer Xylene) mounting medium and covered with glass coverslips. Images were taken using a confocal microscope (FluoView FV 1000; Olympus, Tokyo, Japan). Integrated density was used for the quantification of staining intensity and amount in the immunofluorescent microscopic image. ImageJ software (version 1.50, NIH, https://imagej.nih.gov/ij/, Bethesda, MD, USA) was used to quantify the integrated density which represents the sum of pixel values in an image.

### 2.15. Nissl Staining

All slide sections were washed twice for 5 min in 1% PBS and stained with 0.5% cresyl violet solution including a few drops of glacial acetic acid for 10–15 min, as described previously [34]. All slides were washed with distilled water and dehydrated in graded ethanol (70%, 95%, and 100%), then dried and dipped in xylene for 5 min, and glass coverslips were placed using the mounting medium. The cells in the cortex and hippocampus (DG and CA3) were counted using computerized image analysis.

### 2.16. Fluoro-Jade-B Staining

Fluoro-jade B (FJB) staining was performed according to the manufacturer’s instructions (Cat-AG310, Billerica, MA, USA). Cortex and hippocampus of all mice were dried overnight. All sections were washed twice with 1% PBS for 10 min, followed by immersion in a solution of sodium hydroxide (1% *w*/*v*) and ethanol (80% *v*/*v*) for 5 min. All slides were washed with 70% ethanol followed by distilled water for 2 min, and then dipped in a solution of potassium permanganate (0.06% *w*/*v*) for 10 min and rinsed with distilled water and transferred to a solution of 0.1% acetic acid and 0.01% FJB. After 20 min the slides were washed three times with distilled water and allowed to dry in warm air. Glass coverslips were mounted using DPX nonfluorescent mounting medium. Images were captured using a laser confocal FluoView FV 1000 microscope equipped with FV10-ASW 3.1 viewer (Olympus, Tokyo, Japan) and relative integrated density was used for quantification.

### 2.17. Data and Statistical Analysis

In brief, for Western blot and morphological analysis, ImageJ software (https://imagej.nih.gov/ij/) was used to measure the density of arbitrary units (AU) and integrated density of AU, respectively, and data are expressed as group mean ± standard error of the mean (SEM) of 6–8 animals/group and representative of three independently conducted experiments. For comparison between treatment and control groups, statistical analysis was performed using one-way ANOVA with Tukey’s post hoc test. Statistical significance was determined at *p* < 0.05. 

## 3. Results

### 3.1. Caffeine Ameliorates Cadmium-Induced Cytotoxicity and Elevated ROS/LPO in Vivo and in Vitro

First, we evaluated the effects of different concentrations of caffeine against Cd on cell viability via MTT assay performed on HT-22 cells. The result demonstrated that 20 µM Cd significantly reduced cell viability, and 100 µM caffeine markedly reduced Cd-induced cell toxicity in HT-22 cells (Figure 2a). Next, we performed ROS and LPO assays in vitro on HT-22 cells. Our findings revealed that there was enhanced expression of ROS and LPO in the Cd-alone treated cells compared to the vehicle-treated control group. Interestingly, ROS and LPO levels were significantly downregulated in the Cd + caffeine-treated cells (Figure 2b,c). To confirm these findings in vivo, we examined the detrimental effects of Cd in adult mouse brain (cortex and hippocampus). Interestingly, we found that ROS/LPO levels were significantly elevated in the Cd-alone treated group compared to the vehicle-treated control group. However, ROS/LPO levels were markedly downregulated in the Cd + caffeine-treated group (Figure 2d,e). Overall, the in vivo and in vitro findings support the notion that caffeine reverses the detrimental effects of Cd.

### 3.2. Caffeine Exerts Neuroprotection by Regulating the Expression of Nrf-2/HO-1 Against Cadmium-Induced Oxidative Stress in Vivo

**Figure 3:**
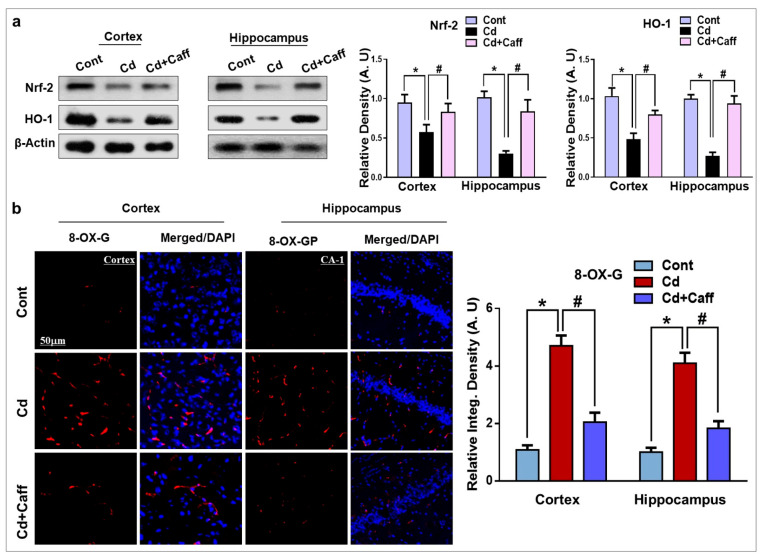
The Cd-induced oxidative stress is ameliorated by caffeine in mice brain. (**a**) The Western blot analysis and relative histograms showing the expression of nuclear factor-2 erythroid-2 (Nrf-2) and hemeoxygenase-1 (HO-1) in the cortex and hippocampus of adult mice. The bands were quantified using ImageJ software, and the differences are represented by histograms. β-actin was used as a loading control. (**b**) The immunoreactivity of 8-dihydro-8-oxoguanine (8-OXO-G) (red) along with their respective histogram stained with 4′,6-diamidino-2-phenylindole dihydrochloride (DAPI) (blue) in cortex and hippocampal Cornu Ammonis-1 (CA1 region) region in the adult mice. The density values are relative to the control (saline-treated) group and expressed in arbitrary units (A.U), with magnification 10×, scale bar = 50 µm. The data are presented as the mean ± SEM of 6–8 mice/group and are representative of the three independent experiments. ⁎ significantly different from vehicle-treated group; # significantly different from the Cd-treated group. Significance = ⁎ *p* < 0.05, # *p* < 0.05.

Extensive studies have reported that Cd is responsible for elevated oxidative stress and neurodegeneration. Nrf-2 plays a protective role against ROS and is also known to detoxify free radicals in the mouse brain. Nrf-2 regulates the expression of HO-1, which has a protective role against oxidative stress and neurodegeneration. To assess the effects of Cd on the expression of Nrf-2 and HO-1, we performed Western blot analysis, which revealed that their expression was significantly downregulated by Cd in the adult mouse brain, and was significantly upregulated in the Cd + caffeine-treated group (Figure 3a). Another main marker used to evaluate oxidative stress, 8-dihydro-8-oxoguanine (8-OXO-G), was assessed in the experimental groups through confocal microscopy. The results revealed that the level of 8-OXO-G was significantly enhanced in the Cd-alone treated group compared to the vehicle-treated control group. Remarkably, the level was significantly downregulated by caffeine (Figure 3b).

### 3.3. The Neuroprotective Effect of Caffeine is Regulated by Nrf-2 Signaling in HT-22 Cells

**Figure 4:**
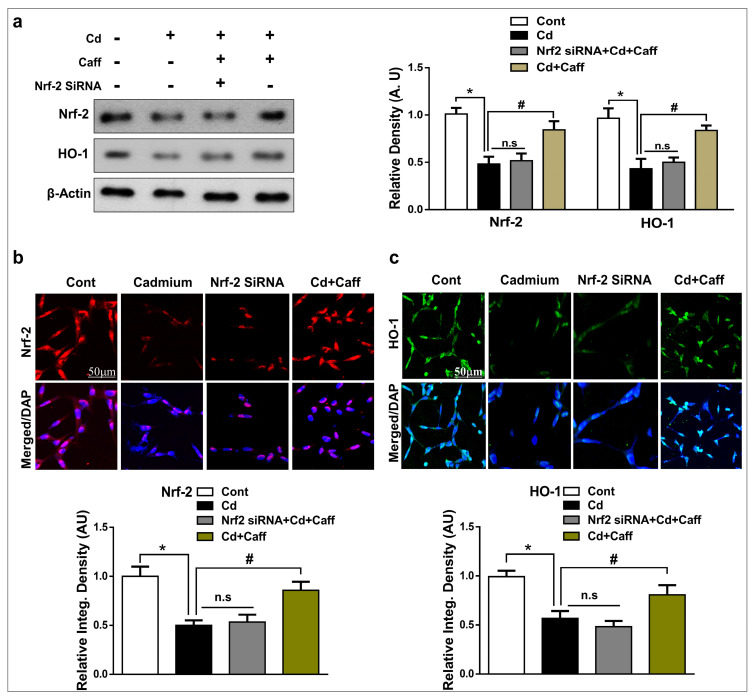
The Cd-induced oxidative stress is ameliorated by caffeine in vitro in HT-22 cells. (**a**) The western blot analysis and relative histograms showing the expression of nuclear factor-2 erythroid-2 (Nrf-2) and hemeoxygenase-1 (HO-1) in the Cd, Caffeine and Nf-2 small interfering RNA (siRNA)-treated HT-22 cells. The bands were quantified using ImageJ software, and the differences are represented by histograms. β-actin was used as a loading control. (**b**) The immunoreactivity of Nrf-2 (red) and HO-1 (green) (**c**) along with their respective histogram stained with DAPI (blue) in HT-22 cells. The density values are relative to the control (vehicle-treated) group and expressed in arbitrary units (A.U.), with magnification 10×, scale bar = 50 µm. The data are representative of the three independent experiments. ⁎ significantly different from vehicle-treated group; # significantly different from the Cd-treated group. Significance = ⁎ *p* < 0.05, # *p* < 0.05.

We further examined the expression of Nrf-2 and HO-1 in vitro by immunoblot and immunofluorescence microscopy to support our in vivo findings. Our results show that Cd treatment significantly reduced the expression of Nrf-2 and its target gene HO-1 in HT-22 cells. On the other hand, the expression of Nrf-2 and HO-1 was significantly restored in the Cd + caffeine-treated group. However, the expression of Nrf-2 and HO-1 was not significantly improved when Nrf-2 genes had been knocked down by Nrf-2 siRNA (Figure 4a,b). Overall, the above findings support the hypothesis that caffeine exerts protective effects against Cd-induced oxidative stress by regulating the Nrf-2/HO-1 signaling pathway.

### 3.4. Caffeine Mitigates Cadmium-Induced Activated Astrocytes and Microglia in the Mouse Brain

**Figure 5:**
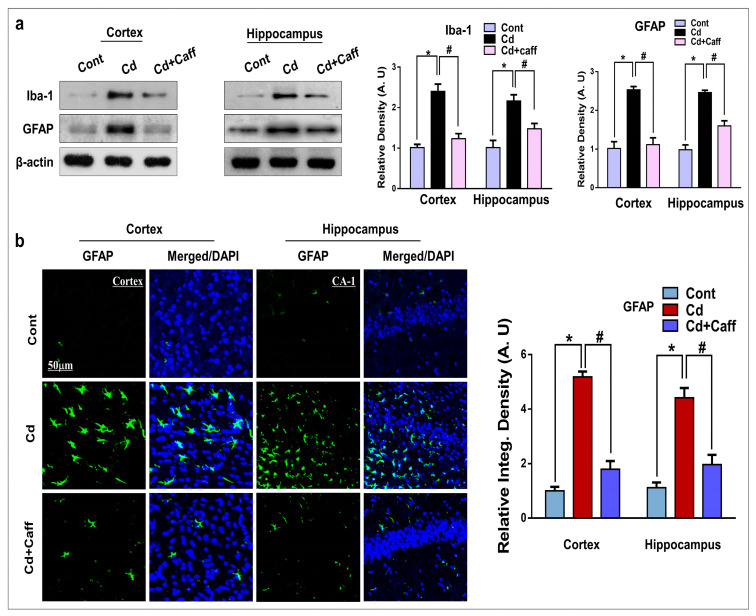
Caffeine attenuates the Cd-activated glial cells. (**a**) Immunoblot analysis of Ionized calcium-binding adaptor molecule 1(Iba-1) and glial fibrillary acidic protein (GFAP) in the cortex and hippocampus of mice (*n* = 6-8 mice/group). The bands were quantified using ImageJ software, and the differences are represented by respective histograms. β-actin was used as a loading control. (**b**) The immunofluorescence analysis of GFAP (green) along with their respective histogram stained with DAPI (blue) in cortex and in the CA1 region of hippocampus in the adult mice. The density values are relative to control (saline-treated) group and expressed in arbitrary units (A.U), with magnification 10×, scale bar = 50 µm. The data are presented as the mean ± SEM of 6–8 mice/group and are representative of the three independent experiments. ⁎ significantly different from the saline-treated group; # significantly different from the Cd-treated group. Significance = ⁎ *p* < 0.05, # *p* < 0.05.

Previous studies have reported that oxidative stress is responsible for the activation of glial cells, which leads to neuroinflammation [35,36]. Ionized calcium-binding adaptor molecule 1(Iba-1) and glial fibrillary acidic protein (GFAP) are the specific markers for activated microglia and astrocytes, respectively. Our Western blot results indicated that Cd-injected mice showed enhanced expression of Iba-1 and GFAP in the cortical and hippocampal regions compared to the control vehicle-treated group (Figure 5a). However, the expression of Iba-1 and GFAP in the Cd + caffeine-treated mice was significantly downregulated compared to the Cd-alone treated mice. Similarly, immunofluorescence revealed that Cd significantly enhanced the immunoreactivity of GFAP compared to the control group, which was significantly downregulated in the Cd + caffeine-treated group compared to the Cd-alone group. (Figure 5b). Overall these results show that caffeine may inhibit the activation of astrocytes and microglia in Cd-treated mice, which may contribute to its anti-inflammatory potential.

### 3.5. Caffeine Suppressed Cadmium-Mediated Release of Inflammatory Cytokines in Vivo and in Vitro

**Figure 6:**
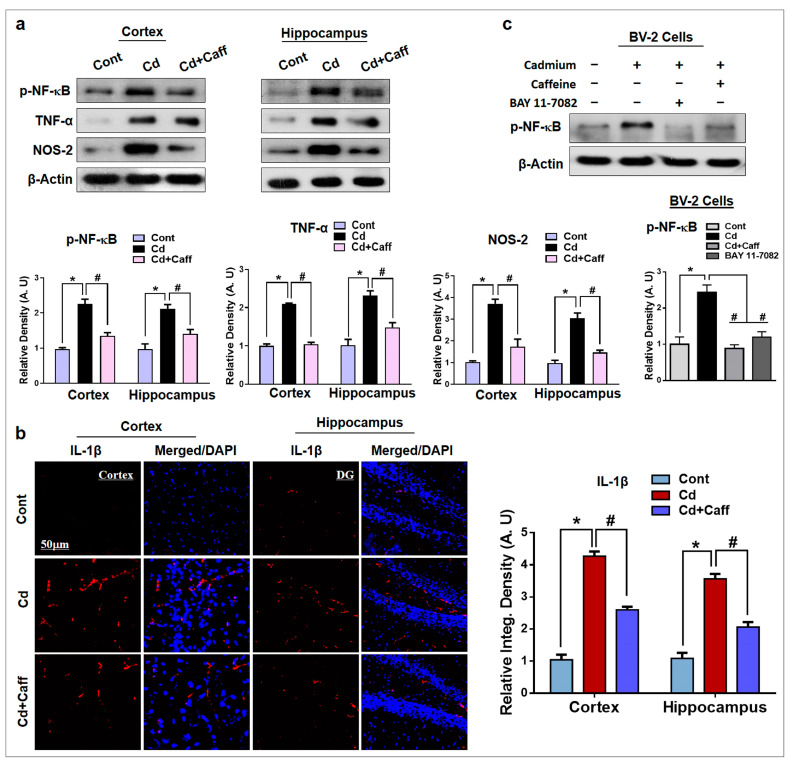
Protective effects of Caffeine on the expression of inflammatory markers. (**a**) The western blot analysis shows the elevated expression of phosphorylated-nuclear factor-κB (p-NF-κB), tumor necrosis factor alpha (TNF-α), nitric oxide synthase type 2 (NOS-2) in the adult mice. The bands were quantified using ImageJ software (version 1.50, NIH, https://imagej.nih.gov/ij/, Bethesda, MD, USA), and the differences are represented by respective histograms. β-actin was used as a loading control. (**b**) Immunofluorescence analysis of interleukin-1β (IL1-β) (red) along with their respective histogram stained with DAPI (blue) in cortex and in the Dentate Gyrus (DG) region of hippocampus in the adult mice. (**c**) The western blot analysis and representative histograms showing the expression of p-NF-κB in the Cd, caffeine, and/or BAY 11-7082-treated BV-2 cells. The bands were quantified using ImageJ software, and the differences are represented by histograms. β-actin was used as a loading control. The density values are relative to the control (vehicle-treated) group and expressed in arbitrary units (A.U), with magnification 10×, scale bar = 50 µm. The data are presented as the mean ± SEM of 6–8 mice/group and are representative of the three independent experiments. ⁎ significantly different from vehicle-treated group; # significantly different from the Cd-treated group. Significance = ⁎ *p* < 0.05, # *p* < 0.05.

It has been reported that Cd is responsible for the activation of several inflammatory markers such as phosphorylated-nuclear factor-κB (p-NF-κB), tumor necrosis factor alpha (TNF-α), nitric oxide synthase type 2 (NOS-2), and interleukin-1β (IL1-β) [37,38]. So, here, we evaluated the expression of p-NF-κB, TNF-α, and NOS-2 in the adult mouse cortex and hippocampus of the experimental groups, which showed Cd-induced upregulation of all three compared to vehicle-treated control mice. Interestingly, the expression of these activated cytokines was significantly downregulated in the Cd + caffeine-treated group compared to the Cd-alone group (Figure 6a). Moreover, we confirmed these findings in vitro in BV-2 cells, which revealed that Cd augmented the p-NF-κB protein level, while caffeine and BAY 11-7082 (an NF-κB inhibitor) reduced this level significantly (Figure 6c). This result supports the hypothesis that the anti-inflammatory effects of caffeine against Cd are mediated via the NF-κB signaling pathway. Similarly, the immunofluorescence results of IL-1β also indicated that Cd administration increased IL-1β immunoreactivity compared to the control group. On the other hand, caffeine significantly downregulated Cd-induced immunoreactivity of IL-1β as compared to the Cd-alone group (Figure 6b). Overall, these results support the hypothesis that caffeine attenuated the Cd-induced release of inflammatory cytokines via NF-κB and thereby rescued the mouse brain against Cd-induced neurodegeneration.

### 3.6. Caffeine Prevents Cadmium-Induced Neuronal Apoptosis and Neurodegeneration in the Adult Mouse Brain 

**Figure 7:**
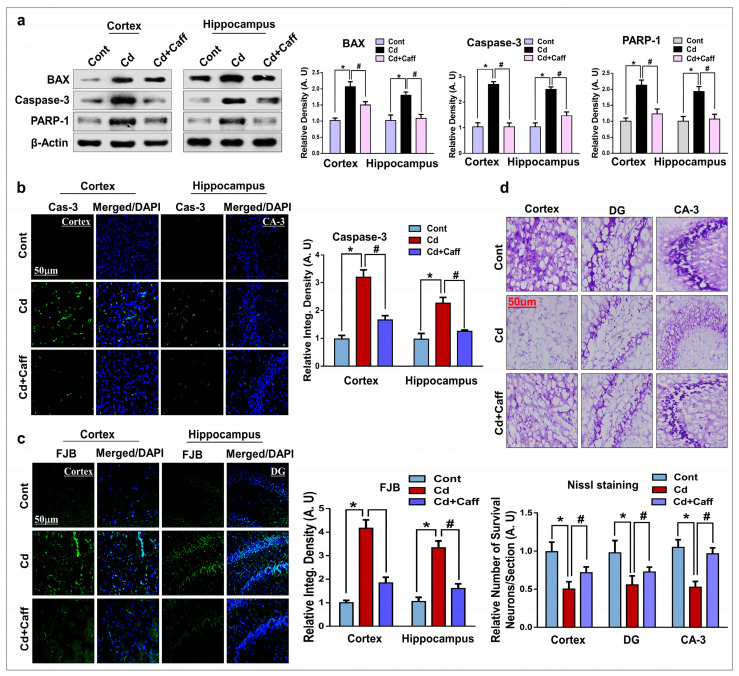
Caffeine attenuated the Cd-induced neuronal apoptosis in the adult mice. (**a**) Western blot analysis of apoptotic markers Bax: (B-cell lymphoma 2)-associated X, caspase-3, and poly (ADP-ribose) polymerase-1 (PARP-1) in the cortex and hippocampus of adult mice. The bands were quantified using ImageJ software, and the differences are shown by their respective histograms. β-actin was used as a loading control. (**b**) Representative images of the immunoreactivity of the caspase-3 in the cortex and Cornu Ammonis-3 (CA-3) region of the hippocampus of adult mice. (**c**) Immunofluorescence analysis of fluoro-jade B (FJB)-positive neurons in the cortex and DG region of the hippocampus of different experimental groups. (**d**) Representative images of the Nissl staining in the cortex, DG and in the CA3 region of hippocampus. The density values are relative to the control (vehicle-treated) group and expressed in arbitrary units (A.U), with magnification 10×, scale bar = 50 µm. The data are presented as the mean ± SEM of 6–8 mice/group and are representative of the three independent experiments. ⁎ significantly different from the saline-treated group; # significantly different from the Cd-treated group. Significance = ⁎ *p* < 0.05, # *p* < 0.05.

Apoptosis, a programmable cell death, is the main feature of acute or chronic neurodegenerative disease. Cd has been shown to be responsible for apoptotic neuronal death [39]. To explore the possible protective effects of caffeine against Cd-induced apoptotic cell death, we analyzed the expression of Bax, caspase-3, and poly (ADP-ribose) polymerase-1 (PARP-1) in the mouse brain. According to our findings, there was enhanced expression of all three in the Cd-treated group as compared to the control saline-treated group, while caffeine significantly downregulated levels as compared to the Cd-alone group (Figure 7a). Moreover, confocal microscopy results showed increased caspase-3 immunoreactivity in the cortex and hippocampus of Cd-injected mouse brain as compared to control vehicle-treated mice. Interestingly, caspase-3 expression was significantly downregulated in the Cd + caffeine-treated group, as indicated by its immunoreactivity (Figure 7b). 

To examine the morphological changes in neurons of the mouse brain, we performed FJB and Nissl analysis. FJB staining showed more dead/damaged neurons in the cortex and hippocampus of Cd-injected mice than in those of the vehicle-treated control group. However, the number of dead neuronal cells was significantly downregulated by caffeine treatment, as visualized by the number of FJB-stained fluorescent neuronal cells (Figure 7c). Nissls staining revealed that Cd significantly reduced the number of live neurons in the cortex and hippocampus regions; however, caffeine significantly reduced the neurotoxicity of Cd (Figure 7d). On the whole, these findings suggest that caffeine significantly downregulated the expression of proapoptotic markers and mitigated the neurodegeneration induced by Cd administration.

### 3.7. Caffeine Significantly Enhanced Synaptic Integrity and Rescued Memory Impairment in Cadmium-Treated Mice

**Figure 8:**
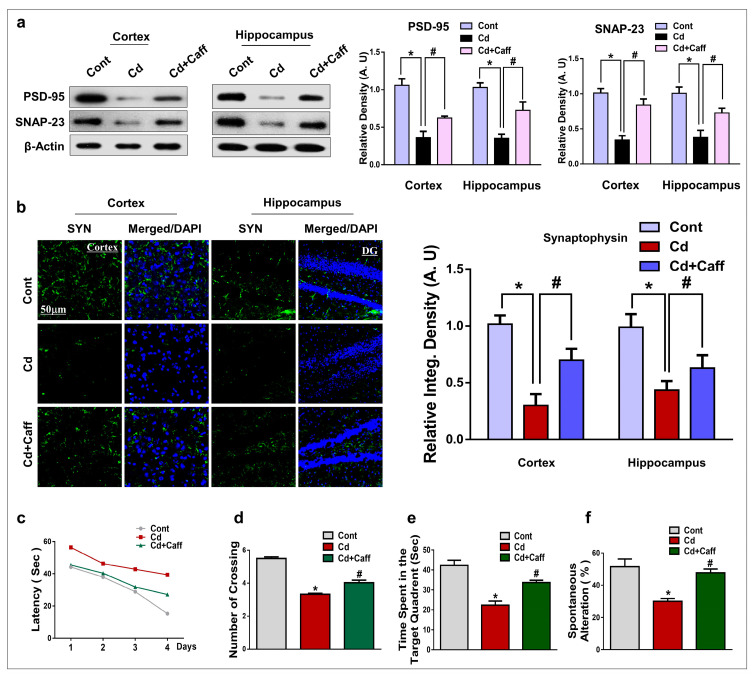
Caffeine enhanced synaptic protein expression and improved cognitive and learning behaviors. (**a**) Western blot analysis of the synaptic postsynaptic density protein-95 (PSD-95) and synaptosomal-associated protein-23 (SNAP-23) protein markers in the cortex and hippocampus of adult mice. The bands were quantified using ImageJ software, and the differences are represented by a histogram. β-actin was used as a loading control. (**b**) Representative immunofluorescence images and the quantified histogram of synaptophysin (SYN) in the cortex and hippocampus of adult mice. (**c**) Average escape latency time is taken by mice to reach the hidden platform (From training day 1 to 4 days). (**d**) An average number of crossing in the under the hidden platform condition during the probe test. (**e**) Time in the target quadrant where the hidden platform was previously placed during the training session. (**f**) Spontaneous alteration behavior % of the mice during the Y-maze test. The density values are relative to the control (vehicle-treated) group and expressed in arbitrary units (A.U), with magnification 10×, scale bar = 50 µm. The data are presented as the mean ± SEM of 6–8 mice/group and are representative of the three independent experiments. ⁎ significantly different from the saline-treated group; # significantly different from the Cd-treated group. Significance = ⁎ *p* < 0.05, # *p* < 0.05.

Previous studies extensively highlighted the downregulation of synaptic proteins in many neurological disorders [40,41]. To evaluate the effects of caffeine against Cd-induced synaptic dysfunction, we examined the relative expression of synaptic proteins. Western blot results indicate that Cd treatment significantly reduced the level of postsynaptic density protein-95 (PSD-95) and synaptosomal-associated protein-23 (SNAP-23), while caffeine significantly improved their expression level in the cortical and hippocampal regions (Figure 8a). Immunofluorescence results revealed that Cd markedly reduced the immunoreactivity of synaptophysin (SYP) as compared to vehicle-treated mice. On the other hand, caffeine treatment significantly improved the immunoreactivity of SYP as compared to Cd-treated mice (Figure 8b).

To assess the effects of caffeine against Cd-induced synaptic dysfunction on cognitive learning and memory processing, we performed Morris water maze (MWM) and Y-maze tasks. After initial training, the latency time in the MWM task was reordered by allowing animals to find and reach the hidden platform. The Cd-alone treated mice took longer to reach the platform compared to the control group, while the latency to reach the hidden platform was significantly reduced in the caffeine-treated group, indicated by less time taken (Figure 8c). A probe test was performed in which the hidden platform was removed, revealing that Cd-injected mice spent less time in the target quadrant, while caffeine-treated mice significantly improved the time spent in the target quadrant as well as the number of times crossing over the previously hidden platform area (Figure 8d,e). Finally, the Y-maze task revealed that Cd prompted short-term spatial memory impairment. Caffeine treatment, on the other hand, considerably enhanced the percentage of spontaneous alternation behavior, which indicates enhancement in the function of spatial working memory (Figure 8f). These results demonstrate that caffeine reversed the detrimental effects of Cd on synaptic integrity, learning, and memory processes.

## 4. Discussion

The goal of the present study was to investigate the antioxidative and neuroprotective effects of caffeine against Cd-induced oxidative stress, neuroinflammation, and neurodegeneration. For the first time we report that the neuroprotective effects of caffeine against Cd-induced neurotoxicity are mediated via Nrf-2/Ho-1 and p-NF-κB signaling regulation. We used caffeine, the world’s most consumed psychostimulant, with the ability to scavenge ROS and boost the antioxidative system, as a neuroprotective agent in this study.

Oxidative stress is an important factor in neurodegenerative disorders, as the brain is the most vulnerable organ to oxidative stress. Cd is a well-known and widespread heavy metal and poison that causes oxidative stress by increasing ROS generation and LPO, and weakens the antioxidative defense system, which ultimately leads to neurodegeneration disorders such as Alzheimer’s and Parkinson’s disease [42,43]. Elevated reactive oxygen species and its accumulation in the brain leads to learning and memory impairment and neurodegeneration. Our data also indicate that Cd treatment markedly promoted ROS and LPO production. However, caffeine substantially reduced ROS and LPO levels in the cortical and hippocampal regions of mice, and in HT-22 cells in vitro (Figure 2b–e). Moreover, several antioxidant pathways are involved in cellular redox homeostasis in which the Nrf-2/HO-1 signaling pathway plays a critical role against oxidative stress [44,45,46]. Nrf-2 is a multifunctional intracellular transcription factor and a key part of the intracellular defense system against free radicals and oxidative stress. Activation of Nrf-2 plays a major role against inflammatory mediators and detoxifying factors, activates antiapoptotic proteins, and has a major role in neuroprotection [44]. Several studies have demonstrated that activation of Nrf-2 targets various genes, specifically HO-1, which exerts protection against oxidative stress, inflammation, and apoptosis and plays a crucial role against neurodegeneration [47,48]. In line with these studies, our findings revealed that significantly decreased expression of Nrf-2 and HO-1 in the cortex and hippocampus of Cd-treated mice. Co-administration of caffeine along with Cd increased Nrf-2 and HO-1 expression in the cortex and hippocampus of adult mice (Figure 3a). Next, we assessed 8-oxoguanine, a well-known marker of elevated oxidative stress, via confocal microscopy and found that Cd significantly increased the immunoreactivity of 8-oxoguanine in the cortex and hippocampus, while caffeine markedly reduced its immunoreactivity (Figure 3b). To further confirm that caffeine exerts neuroprotective effects by regulating Nrf-2 and HO-1 expression, we knocked down the Nrf-2 genes by Nrf-2-siRNA in HT22 cells. Our immunoblot results show that caffeine treatment did not increase Nrf-2 and HO-1 expression in Cd-exposed HT-22 cells in which the Nrf-2 genes had been knocked down (Figure 4a). Our immunofluorescent results also support the immunoblot results. These findings show that caffeine protects against oxidative stress by upregulating the master endogenous antioxidant Nrf-2/HO-1 pathways (Figure 4b,c). 

Excessive production of ROS damages the DNA, other proteins, and lipids, which further activates genes involved in the inflammatory pathway. Chronic neuroinflammation plays an important role in the pathophysiology of neurodegeneration [49,50]. Astrocytes and microglia in the CNS are part of the immune system, and activated astrocytes and microglia are involved in neuroinflammation and neurodegeneration [25,40,51,52]. We found that the expression of glial fibrillary acidic protein (GFAP), a specific marker for astrocytes, and ionized calcium binder adaptor protein-1 (Iba-1), a specific marker for microglia, were significantly enhanced by Cd. On the contrary, caffeine substantially downregulated GFAP and Iba-1 expression (Figure 5a,b). Moreover, NF-κB, a pleiotropic transcription factor, regulates different genes associated with the inflammatory response and stimulates several cellular pathways, which results in enhanced release of proinflammatory mediators. Cd-induced NF-κB overexpression in astrocytes and microglia has been reported to upregulate the expression of inflammatory mediators [53,54,55,56]. Furthermore, tumor necrosis factor alpha (TNF-α) is a pro-inflammatory cytokine and its overexpression is important in the initiation of neuroinflammation and neurodegeneration. Interleukin-1β (IL-1 β) and nitric oxide synthase type 2 (NOS-2) are also major pro-inflammatory cytokines in the brain and main players in neuroinflammation [35,57,58]. In line with those previous studies, our data indicate that Cd is responsible for overexpression of p-NF-κB, TNF-α, and NOS-2 in the cortex and hippocampus of adult mouse brain; however, treatment with caffeine reduced its expression (Figure 6a,b). Again, we assessed whether this anti-inflammatory potential is mediated by NF-κB. Thus, we treated with caffeine and BAY 11-7082 together with Cd in BV-2 cells. Interestingly, caffeine and BAY 11-7082 reduced the p-NF-κB level substantially after Cd treatment. These results suggest that the effect of caffeine on neuroinflammation is markedly induced by the NF-κB signaling pathway, consistent with the specific NF-κB inhibitor (Figure 6c).

Previous studies have demonstrated that Cd-induced oxidative stress activates various neuronal apoptotic proteins, which ultimately leads to neuronal cells death [59,60]. During apoptosis, Bax translocates from the cytosol to mitochondria, which activate mitochondrial-dependent apoptosis, followed by cytochrome-c release, mitochondrial dysfunction, and caspase activation, which ultimately leads to cell death [61,62,63]. Caspase-3 is recognized as an important feature of apoptosis in neuronal cells. Activation of caspase-3 results in neuronal cell death and is also a main feature of neurodegenerative disease [64,65,66]. Activated caspase-3 cleaves poly (ADP-ribose) polymerase-1 (PARP-1), which acts against the repair of damaged DNA and results in energy deprivation of cells, ultimately leading to apoptotic cell death [67,68,69]. The present study also demonstrates that Cd significantly enhanced Bax, caspase-3, and PARP-1 levels, while caffeine significantly reduced their levels. Also, FJB staining, a histological analysis used for degenerative neurons [70], demonstrates that caffeine significantly attenuated the detrimental effects of Cd by reducing the number of FJB-stained neurons in the hippocampus and cortex. Nissl staining result also support the finding that caffeine significantly enhanced the survival of neurons (Figure 7a–d).

Chronic exposure to Cd interferes with synaptic transmission and synaptic plasticity, which leads to neurological disorders such as olfactory dysfunction and Parkinson’s disease [71]. We also found that Cd markedly reduced the expression of synaptic proteins (PSD-95, SNAP-23, and SYN) in the cortex and hippocampus of Cd-treated mice, and administration of caffeine significantly enhanced its expression. Furthermore, studies have reported that Cd is neurotoxic and exposure to Cd is responsible for impaired cognition and memory in several animal models [72,73]. We performed behavioral analysis (MWM and Y-Maze) to measure spatial learning and memory function. Our results show that Cd is responsible for a significant reduction in spatial cognition and memory functions. Administration of caffeine significantly improved memory functions (Figure 8). 

## 5. Conclusions

On the whole, these findings reveal that caffeine ameliorated the Cd-induced neuropathological hallmarks in vivo (in adult mouse brains) and in vitro (in HT-22 and BV-2 cells). Our data suggest that caffeine attenuates Cd-induced oxidative stress-mediated neuroinflammation, neurodegeneration, cognitive decline, and memory impairment. However, more comprehensive studies are required to evaluate the mechanistic role of caffeine in oxidative stress and neurodegenerative disorders.

## Figures and Tables

**Table 1:** List of primary antibodies and their information used in this study.

Antibody	Host	Application (Conc.)	Manufacturer	Catalog #
Nrf-2	Rabbit	WB/IF (1:1000/1:100)	Santa Cruz Biotechnology (Dallas, TX, USA)	SC 722
HO-1	Mouse	WB/IF (1:1000/1:100)	Santa Cruz Biotechnology (Dallas, TX, USA)	SC 136961
8-OXO-G	Mouse	IF (1:100)	Millipore, USA (Billerica, MA, USA)	MAB3560
Iba-1	Rabbit	WB (1:1000)	Santa Cruz Biotechnology (Dallas, TX, USA)	SC 98468
GFAP	Mouse	WB/IF (1:1000/1:100)	Santa Cruz Biotechnology (Dallas, TX, USA)	SC 33673
p-NF-κB	Mouse	WB (1:1000)	Santa Cruz Biotechnology (Dallas, TX, USA)	SC 136548
TNF-α	Mouse	WB (1:1000)	Santa Cruz Biotechnology (Dallas, TX, USA)	SC 8436
NOS-2	Rabbit	WB (1:1000)	Santa Cruz Biotechnology (Dallas, TX, USA)	SC 651
IL-1β	Mouse	IF (1:100)	Santa Cruz Biotechnology (Dallas, TX, USA)	SC 32294
Bax	Mouse	WB (1:1000)	Santa Cruz Biotechnology (Dallas, TX, USA)	SC 7480
Caspase-3	Mouse	WB/IF (1:1000/1:100)	Santa Cruz Biotechnology (Dallas, TX, USA)	SC 7272
PARP-1	Mouse	WB (1:1000)	Santa Cruz Biotechnology (Dallas, TX, USA)	SC 8007
PSD-95	Mouse	WB (1:1000)	Santa Cruz Biotechnology (Dallas, TX, USA)	SC 71933
SYP	Mouse	IF (1:100)	Santa Cruz Biotechnology (Dallas, TX, USA)	SC 17750
SNAP-23	Mouse	WB (1:1000)	Santa Cruz Biotechnology (Dallas, TX, USA)	SC 374215

WB: Western blot; IF: immunofluorescence; Nrf-2: nuclear factor-2 erythroid-2; HO-1: hemeoxygenase-1; 8-OXO-G: 8-dihydro-8-oxoguanine; GFAP: glial fibrillary acidic protein; Iba-1: ionized calcium-binding adapter molecule 1; p-NF-κB: phosphorylated-nuclear factor-κB; TNF-α: tumor necrosis factor alpha; NOS-2: nitric oxide synthase type 2; IL-1β: interleukin-1β; Bax: (B-cell lymphoma 2)-associated X; PARP-1: polymerase-1; PSD-95: postsynaptic density protein-95; SNAP-23: synaptosomal-associated protein-23; SYP: synaptophysin; #: number.
