# Correction: Khan et al. Caffeine Modulates Cadmium-Induced Oxidative Stress, Neuroinflammation, and Cognitive Impairments by Regulating Nrf-2/HO-1 In Vivo and In Vitro. *J. Clin. Med.* 2019, *8*, 680

**DOI:** 10.3390/jcm14186426

**Published:** 2025-09-12

**Authors:** Amjad Khan, Muhammad Ikram, Tahir Muhammad, Junsung Park, Myeong Ok Kim

**Affiliations:** Division of Applied Life Science (BK 21), College of Natural Sciences, Gyeongsang National University, Jinju 52828, Republic of Korea; amjadkhan@gnu.ac.kr (A.K.); qazafi417@gnu.ac.kr (M.I.); mtahir.khan@gnu.ac.kr (T.M.); jsp@gnu.ac.kr (J.P.)

## Error in Figure

In the original publication [1]. There was a mistake in Figure 3a (hippocampus) and Figure 6a (cortex) regarding β-actin bands. As these two projects were running simultaneously, some data may have been shuffled or combined during the arrangement process which we didn’t notice at that time.

We have reanalyzed and updated the β-actions of both figures, which is shown below. The scientific conclusions of the manuscript are unaffected by updating the β-actin bands.

The corrected Figure 3 appears below.

**Figure 3:**
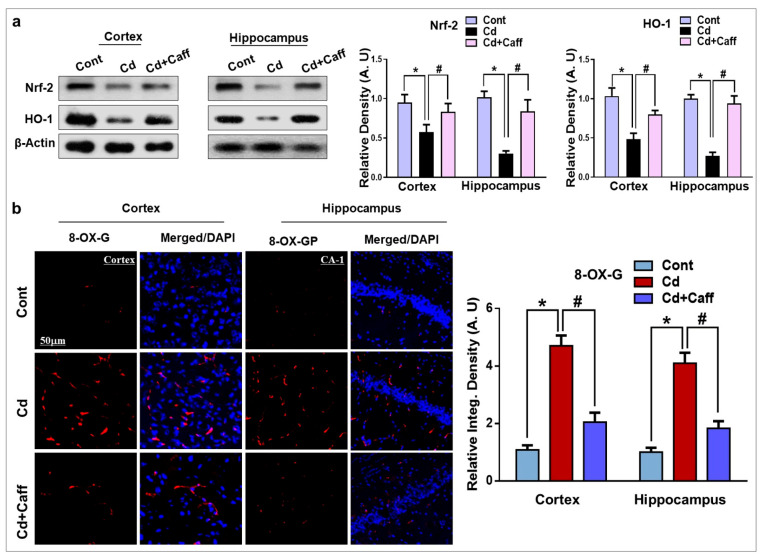
The Cd-induced oxidative stress is ameliorated by caffeine in mice brain. (**a**) The Western blot analysis and relative histograms showing the expression of nuclear factor-2 erythroid-2 (Nrf-2) and hemeoxygenase-1 (HO-1) in the cortex and hippocampus of adult mice. The bands were quantified using ImageJ software, and the differences are represented by histograms. β-actin was used as a loading control. (**b**) The immunoreactivity of 8-dihydro-8-oxoguanine (8-OXO-G) (red) along with their respective histogram stained with 4′,6-diamidino-2-phenylindole dihydrochloride (DAPI) (blue) in cortex and hippocampal Cornu Ammonis-1 (CA1 region) region in the adult mice. The density values are relative to the control (saline-treated) group and expressed in arbitrary units (A.U), with magnification 10×, scale bar = 50 µm. The data are presented as the mean ± SEM of 6–8 mice/group and are representative of the three independent experiments. ⁎ significantly different from vehicle-treated group; # significantly different from the Cd-treated group. Significance = ⁎ *p* < 0.05, # *p* < 0.05.

The corrected Figure 6 appears below.

**Figure 6:**
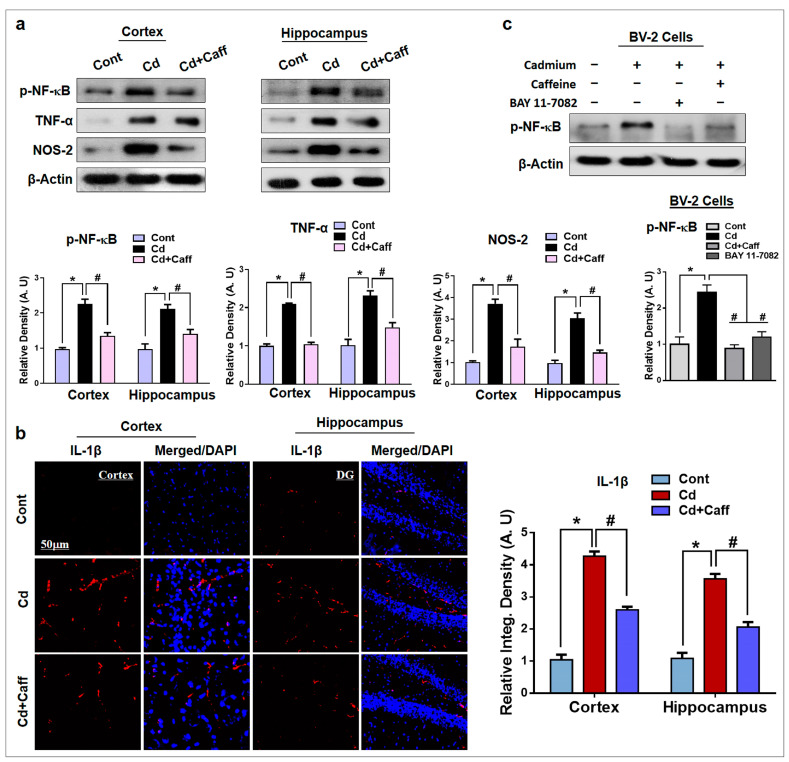
Protective effects of Caffeine on the expression of inflammatory markers. (**a**) The western blot analysis shows the elevated expression of phosphorylated-nuclear factor-κB (p-NF-κB), tumor necrosis factor alpha (TNF-α), nitric oxide synthase type 2 (NOS-2) in the adult mice. The bands were quantified using ImageJ software (version 1.50, NIH, https://imagej.nih.gov/ij/, Bethesda, MD, USA), and the differences are represented by respective histograms. β-actin was used as a loading control. (**b**) Immunofluorescence analysis of interleukin-1β (IL1-β) (red) along with their respective histogram stained with DAPI (blue) in cortex and in the Dentate Gyrus (DG) region of hippocampus in the adult mice. (**c**) The western blot analysis and representative histograms showing the expression of p-NF-κB in the Cd, caffeine, and/or BAY 11-7082-treated BV-2 cells. The bands were quantified using ImageJ software, and the differences are represented by histograms. β-actin was used as a loading control. The density values are relative to the control (vehicle-treated) group and expressed in arbitrary units (A.U), with magnification 10×, scale bar = 50 µm. The data are presented as the mean ± SEM of 6–8 mice/group and are representative of the three independent experiments. ⁎ significantly different from vehicle-treated group; # significantly different from the Cd-treated group. Significance = ⁎ *p* < 0.05, # *p* < 0.05.

The authors state that the scientific conclusions are unaffected. This correction was approved by the Academic Editor. The original publication has also been updated.
